# A Non-Invasive Stress Assay Shows That Tadpole Populations Infected with *Batrachochytrium dendrobatidis* Have Elevated Corticosterone Levels

**DOI:** 10.1371/journal.pone.0056054

**Published:** 2013-02-13

**Authors:** Caitlin R. Gabor, Matthew C. Fisher, Jaime Bosch

**Affiliations:** 1 Department of Biology, Texas State University, San Marcos, Texas, United States of America; 2 Department of Infectious Disease Epidemiology, Imperial College London, Norfolk Place, London, United Kingdom; 3 Museo Nacional de Ciencias Naturales, Consejo Superior de Investigaciones Científicas, Madrid, Spain; Instituto Butantan, Brazil

## Abstract

*Batrachochytrium dendrobatidis* (*Bd*) is a fungus that causes the disease chytridiomycosis and is associated with widespread amphibian declines. Populations vary in their susceptibility to *Bd* infections, and the virulence of the infecting lineage can also vary. Both of these factors may manifest as a differential physiological stress response. In addition, variation in disease susceptibility across amphibian populations may be influenced by immunosuppression caused by chronic stress imposed by environmental factors. Here, we use a non-invasive water-borne hormone technique to assess stress levels (corticosterone) of free-living tadpole populations that are infected by *Bd*. We found that corticosterone release rates were higher in infected populations of two species of tadpoles (*Alytes obstetricans* and *A*. *muletensis*) than in an uninfected population for both species. The relationship between corticosterone and the intensity of infection differed between species, with only the infected *A. obstetricans* population showing a significant positive correlation. The higher corticosterone release rates found in *A. obstetricans* may be an outcome of infection by a highly virulent lineage of *Bd* (*Bd*GPL), whereas *A. muletensis* is infected with a less virulent lineage (*Bd*CAPE). These results suggest that different lineages of *Bd* impose different levels of stress on the infected animals, and that this may influence survival. The next step is to determine whether higher corticosterone levels make individuals more susceptible to *Bd* or if *Bd* infections drive the higher corticosterone levels.

## Introduction

*Batrachochytrium dendrobatidis* (*Bd*) is an aquatic chytrid fungus that causes the globally emerging disease chytridiomycosis in amphibians [1]–[3]. The pathogen infects host skin and innate cutaneous immune defenses constitute frontline protection against infection. Specifically, the production of skin antimicrobial peptides and inflammatory reactions appear to be the main routes of defense against *Bd,* which may include a microbial component [4], [5]. It is also known that environmental stressors impair host-produced defenses against *Bd* [5], [6], leading to higher burdens of infection. However, the mechanisms underpinning the interaction between environmental assaults and amphibian immunity are not well understood [6].

The amphibian immune response to infection is modulated by the hypothalamus-pituitary-interrenal (HPI) axis [7]. Glucocorticoids, the main vertebrate stress hormones, are released within 10 min of stress-induced activation of the HPI but generally actions are not exerted for about an hour after the onset of the stressor [8]. Initial release of corticosterone (CORT), the main amphibian glucocorticoid, may initially activate the immune system [9]. Over longer periods of time (hours or days) with chronic exposure to a stressor, as CORT levels rise, immune cell function becomes inhibited and acts as a negative feedback on the HPI resulting in decreased growth, development and immunosuppression. When confronted by infection, these factors can result in increased susceptibility to disease [6].

In amphibians, elevated CORT can accelerate metamorphosis at the cost of additional immune responses [10], [11]. Several studies on frogs have examined aspects of the immune and glucocorticoid responses to infection and found higher circulating leukocytes [12] and elevated CORT (urinary) profiles in infected individuals [13]. Additionally, tadpoles infected with ranavirus have elevated whole-body CORT and elevated CORT drives faster metamorphosis at a lower body mass [10]. Poor condition at metamorphosis and the additional costs of responding to a pathogen can decrease the probability of survival in amphibians [14], [15]. In sum, sustained CORT levels during tadpole development may suppress immune response to *Bd* and may increase suboptimal timing of metamorphosis, which eventually contributes to greater likelihood of death.

Populations of amphibians vary in their susceptibility to infection by *Bd* and this differential response owes to both host- and pathogen-dependent components [6], [14], [16], [17]. Knowledge of the physiological mechanisms that mediate susceptibility to infection may provide insights into why populations vary in infection levels and why some are extirpated and others persist[18]. However, the link between physiology and *Bd* infection is difficult to establish especially in free-living amphibians [19]. A limitation to physiological studies in small amphibians is that they require euthanizing in order to obtain sufficient plasma to measure hormones. Here we use a non-invasive water-borne hormone collection method that was developed for fish [20], which assesses free steroids that passively diffuse from the bloodstream into the water through the gills, urine, and feces [21]. The correlation between CORT water-borne hormone release rates and CORT plasma levels has been validated for *Alytes obstetricans* (common midwife toad) [22].

We hypothesized that CORT in tadpoles will vary with *Bd* infection. We also predicted that populations infected with *Bd* would have higher CORT levels than those without. We tested these predictions in free-living populations of two closely related species of toads: *Alytes obstetricans* (common midwife toad) and the IUCN red-listed Mallorcan midwife toad *(A. muletensis*).

*Alytes obstetricans* are highly susceptible to *Bd* [23] as is its close congener *A. muletensis* [24]. Mortality primarily affects recently metamorphosed individuals and *Alytes* populations with *Bd* infected metamorphs exhibit differential rates of mortality across their range [18], [25]. To assess whether infection by *Bd* is associated with increased stress levels, we measured water-borne CORT release rates of individual tadpoles in the field for one uninfected and one infected population for each species of *Alytes*.

## Materials and Methods

### Study Sites and Samples

Tadpoles of *Alytes obstetricans* and *A. muletensis* from four field sites were collected on 7–14 May 2011. We sampled *A. obstetricans* tadpoles at the currently uninfected population at Circo del Nevero, Guadarrama Mountain, Central Spain (40.98 N, −3.84 W) and an infected population in Toro, Northwest Spain (41.48 N, −5.45 W). These sites were not privately owned or protected. We also sampled tadpoles of *A. muletensis* at one uninfected and one infected population (on private property) in Serra de Tramuntana, North Mallorca (39.88 N, 2.86 W and 39.81 N, 2.73 W respectively). The owners provided access to the private Mossa property in Mallorca. Animal welfare was approved by the Consejerias de Medio Ambiente of Madrid (permit 10/121009.9/11), Castilla y León (ISA/pa EP/CYL/54/2011 and Illes Balears (CAP41/2011).

At each population we used a dip net to collect tadpoles (n = 20 for each population) and then placed individual tadpoles in clean, uninfected 100 ml beakers (cleaned with 99% ethanol and rinsed with DI water) filled with 40 ml of water (treated tap water or pond water) for one hour to collect water-borne hormones. Gloves were worn throughout the process. Due to the distance travelled to get to the *A. muletensis* populations, we used pond water to assay hormones. Therefore, we also saved a sample of the pond water from those populations and assayed their levels of CORT. Contents of the beaker were poured through a course sieve into Falcon tubes. Before releasing tadpoles back into their pond, the keratinized mouthparts of tadpoles were swabbed using sterile cotton-tipped swabs (MW100; Medical Wire & Equipment Co, Corsham, UK) for later diagnostic quantitative PCRs. We also measured the snout vent length (svl) of tadpoles and recorded their approximate Gosner stage [26]. All tadpoles were sampled between 1100 h–1400 h to minimize effects of circadian variation in CORT. Samples were placed on ice in coolers and transported back to the laboratory. No specific permits were required for any of the other described field studies including the ‘water’ collections. Water-borne hormone samples were stored at −20°C until ready to be thawed for extraction. After extracting hormones from water using C 18 solid phase extraction columns (SEP-PAK, Waters Inc.) the columns were stored at −20°C for methanol elution at Texas State University following [27]. The methanol was evaporated under a gentle stream of nitrogen gas while placed in a 37°C water bath. We re-suspended the residue in 5% ethanol followed by vortexing for 1 m and then 95% EIA buffer (provided by Cayman Chemicals Inc. Ann Arbor, MI, USA) for a final re-suspension volume of 250 µl. We diluted samples to 1∶14 with EIA buffer. Corticosterone hormone levels were measured using an enzyme-immunoassay (EIA) kit (Cayman Chemicals Inc.). We subtracted the CORT level measured in the pond water from each of the individual CORT measures for the tadpoles from the respective populations from which we used pond water. The *Bd* infected *A. muletensis* population had 120.94 pg of CORT in the pond water (following prior protocol we multiplied the EIA CORT assay output by our re-suspension volume so the unit of CORT is just pg) and the uninfected population had 72 pg of CORT in the pond water.

### Water-borne Hormone Methods

Gabor et al. [22] validated that water-borne CORT release rates are a predictor of plasma CORT levels for *A. obstetricans*. Further, Gabor et al. [22] validated the use of the CORT EIA kits with water-borne hormones for *A. obstetricans*. For this paper, we additionally validated the use of the CORT EIA kits with water-borne hormones for *A. muletensis*. The unmanipulated pooled controls (from 10 non-experimental *A. muletensis* tadpoles) were diluted to 1∶2 for the serial dilutions and quantitative recovery. We ran serial dilution of the pooled controls in duplicate. The serial dilution curve was parallel to the standard curve (comparison of slopes: t_9_ = 1.32, P = 0.22). To examine recovery, we cold spiked equal volumes of the pooled controls with each of the eight standards. The expected recovery concentrations were based on the known amount of CORT in the unmanipulated pool control sample. The minimum observed recovery for *A. muletensis* was 63%. The regression coefficient of the observed vs. expected concentrations was 1.04 (F_1,8_ = 559.5, r^2^ = 0.99; P<0.0001).

### Quantitative PCR Methods

Larval mouthparts were screened for *Bd* infection with a quantitative real-time polymerase chain reaction (qPCR) protocol [28]. Nucleic acids were extracted from samples following [28], and extractions were diluted 1∶10 before real-time PCR amplification was performed in duplicate, with *Bd* genomic equivalent (GE) standards of 100, 10, 1, and 0.1 GE. When only one replicate from any sample amplified, we ran this sample a third time. If the third amplification did not result in an amplification profile, we considered the sample negative for infection.

### Statistical Analyses

We normalized the hormone data by dividing by the snout vent length (svl) of the tadpoles. In both species of tadpoles there is a strong positive relationship between svl and mass (Linear regression: *A. obstetricans*: r^2^ = 0.85, n = 21; P* = *0.0001: *A. muletensis:* r^2^ = 0.95, n = 30; P* = *0.0001). The Ln transformed normalized hormone data met the assumptions of parametric analyses so we used unpaired Student’s *t*-test. We were unable to transform the zoospore count (GE) data to meet the assumptions of parametric statistics so we used a generalized linear model (GLM) and a Wilcoxon test when examining the data related to GE values. One sample was lost due to spills or problems with extracting hormones for each population (except for the infected *A. obstetricans* population). We used two tailed *p* values and alpha was set at 0.05.

## Results

Uninfected populations of *A. obstetricans* and *A. muletensis* were confirmed negative for infection by qPCR. In the infected populations the intensities of infection (zoospore count GE) ranged from 0 (2 cases)–1780 GE (mean ± S.E. GE was 371.45±105.16) for *A. obstetricans* and 0 (five cases)-171 GE (mean ± S.E. GE was 42.82±10.65) for *A. muletensis*. The mean SVL for the uninfected *A. obstetricans* (± S.E. mm) was 20.32 (±0.50) and *A. muletensis* was 16.35 (±0.71). The mean SVL for the infected *A. obstetricans* (± S.E. mm) was 22.90 (±0.59) and *A. muletensis* was 18.05 (±0.47). The mean Gosner stage for the uninfected *A. obstetricans* (± S.E.) was 33.42 (±0.14) and *A. muletensis* was 31.95 (±0.37). The mean Gosner stage for the infected *A*. *obstetricans* was 33.75 (±0.23). All but one of the infected *A. muletensis* was at Gosner stage 33 and the other was at 32.

**Figure 1:**
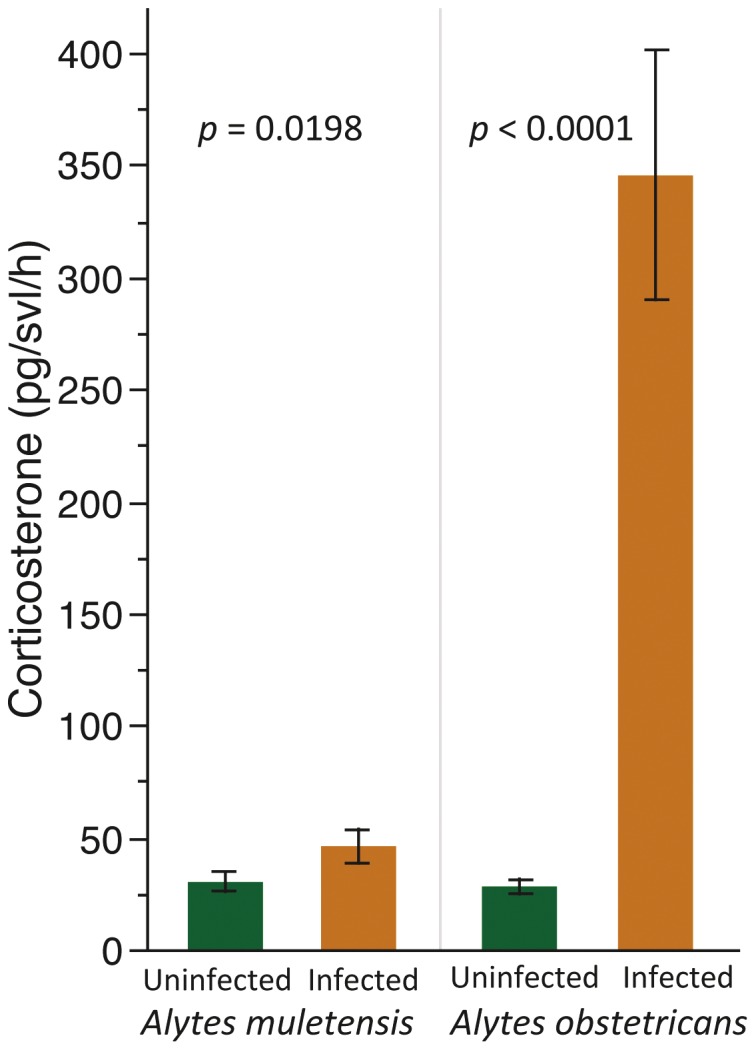
Mean corticosterone release rates. The mean corticosterone (pg/svl/h) ± S.E. of infected vs uninfected populations of *Alytes obstetricans* and *A*. *muletensis*.

**Figure 2:**
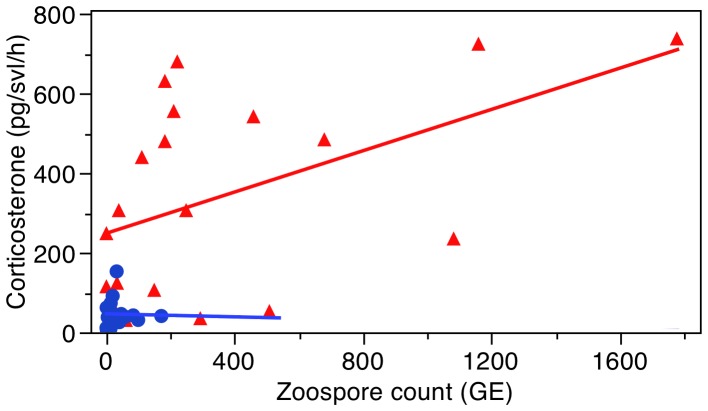
Correlation between corticosterone release rates and zoospore count. The correlation between corticosterone levels (pg/svl/h) and *Batrachochytrium dendrobatidis* zoospore count (GE) for *Alytes obstetricans* (red triangles) and *A. muletensis* (blue circles).

We found that the infected population of each species had higher CORT release rates (pg/svl/h) than the uninfected population (Unpaired t-test: *A. obstetricans:* t = 8.29, d.f. = 37, P<0.0001; *A. muletensis:* t = 2.44, d.f. = 36, P = 0.019; Figure 1). For *A. obstetricans* there was a significant positive correlation for CORT release rates with zoospore count (GLM: X^2^ = 3.71, P = 0.05; Figure 2) but not with Gosner stage (GLM: X^2^ = 1.03, P = 0.31). There was no interaction between zoospore count and Gosner stage. For *A. muletensis* there was no significant correlation for CORT release rate with zoospore count (GLM: X^2^ = 0.47, P = 0.49; Figure 2) or with Gosner stage (GLM: X^2^ = 0.000009, P = 0.99). There was no interaction between zoospore count and Gosner stage.

We compared the CORT release rates and zoospore counts between the two species to explore the basis of the different relationships between CORT and the intensity of infection: the infected population of *A. obstetricans* had significantly higher CORT than the infected population of *A. muletensis* (Unpaired Student’s t-test: t* = *6.23, d.f. = 37, P<0.0001; Figure 1) but there was no difference in the CORT levels of the uninfected populations of the two species (t = 1.124, d.f. = 36, P = 0.269; Figure 1). The zoospore count was significantly less in the infected *A. muletensis* vs *A. obstetricans* population (Wilcoxon test: z = 3.43, n = 20, P = 0.0006).

## Discussion

Our results demonstrate that infected free-living populations of two species of *Alytes* tadpoles had elevated CORT release rates compared to their respective uninfected population. These data support our hypothesis that infection leads to a chronic stress response, which along with accelerating metamorphosis [11], may lead to decreased survival [14], [15]. Our results are in agreement with other studies that examined the relationship between disease and stress [10], [12], [13]. What is yet to be resolved is whether the HPI axis is activated in response to infection or if the interaction is bidirectional. It is also unclear whether immunosuppression from chronic stress leads to increased susceptibility to infection by *Bd.*

Our study made two significant observations: (1) CORT release rates and GE values were significantly higher in the infected population of *A. obstetricans* compared to *A. muletensis*, and (2) while there was a significant positive correlation between CORT release rates and the intensity of infection in *A. obstetricans*, this relationship was not observed in *A. muletensis.* So, what may be driving these patterns and do they matter?

There are at least three lineages of *Bd*: *Bd*GPL; *Bd*CAPE and *Bd*CH [16]. *Bd*GPL is the hypervirulent, invasive lineage that is associated with major epizootics in Australia, the Americas, and Europe, and is hypervirulent when compared against *Bd*CAPE. *Bd*GPL widely infects mainland Iberian populations of amphibians [25] and is associated with mass-mortality events and population extirpations. In comparison, *Bd*CAPE infects only Mallorcan *A. muletensis* owing to an inadvertent introduction event from a hypothesized point of origin in Africa [29]. Therefore, these two species of *Alytes* are infected by lineages of *Bd* with very different genotypes, phenotypes, morphology and virulence [24]. In this case, the low release rates of CORT in *A. muletensis* may owe to these animals being infected with a pathogen that is intrinsically less virulent, and hence imposes less stress, to the infected animals. In contrast, *A. obstetricans* is infected with the most virulent lineage that may explain the elevated CORT release rates, and thus drive the correlation between *Bd* and CORT. This hypothesis awaits further testing using *in vivo* controlled infections of *Alytes* with different lineages. Additionally, administering an adrenocorticotropic hormone (ACTH) challenge would allow us to determine if the HPI axis is non-responsive. This would demonstrate that the observed CORT levels in *A. muletensis* are lower because they are dampened possibly due to chronic stress.

Our data also do not allow us to determine how CORT responses affect survival due to *Bd* infections. Populations that mount stronger CORT responses may survive *Bd* infections better. Alternatively, populations with stronger CORT responses may be more susceptible to *Bd* owing to a stress-induced immunosuppression. CORT is generally elevated during metamorphosis [30], [31] and tadpoles with elevated CORT that were infected with ranavirus metamorphosed quicker with lower body mass [10]. Tadpoles with lower CORT levels might survive and metamorphose better than those with elevated CORT because elevated CORT inhibits the renewal of amphibian skin peptides [6]. It is now necessary to determine whether approaching mortality results in a systemic stress response, or whether elevated levels of CORT are in direct response to infection by *Bd*.

One limitation of this study was that we were unable to sample numerous infected and uninfected populations of each species. It is possible that other environmental variables such as temperature, community composition and predation intensity could have caused these differences. However, the existence of a correlation between *Bd* load and CORT in the *Bd*GPL infected *A. obstetricans* population (Figure 2) suggests that *Bd* and not other environmental variables are driving this relationship. With that said, the two highest CORT values may be driving this correlation and so the correlation should be considered with caution. We do not think the increase in CORT is an outcome of greater CORT levels in the water. Glennenmeier and Denver [32] found no change in whole body CORT after 18 days of exposure to 62 nM ( = ∼20.8 ng/ml) of exogenous CORT in their water. Nonetheless, the pattern of increased CORT in *Bd* infected populations is observed in both species suggesting that these results are not coincidental. Some variation in susceptibility to *Bd* may also have a genetic basis affecting amphibian tolerance or resistance to *Bd* infection [18] but this alone cannot account for the significant differences in CORT release rates between infected and uninfected populations.

We do not think that our results are an outcome of the stress response due to capture, handling and restraint in the container for two reasons. First, we also obtained CORT values for an uninfected population of *A. muletensis* in captivity (Marineland, Mallorca) during the same dates and time as the other data were obtained. There was no significant difference in the CORT values of the captive population compared to the uninfected field population of this species (Unpaired t-test: t = 1.65, d.f. = 28, P = 0.11; mean ± S.E. pg/svl/h = Field population: 24.63±4.23; lab population: 39.63±8.74). These results suggest that the process of capture is not causing the differences in CORT values because CORT did not differ between a field caught sample and easily caught laboratory tadpoles. Moreover, if this process were causing high stress levels, then we would not expect to find differences between the populations as methods of capture were the same between sites.

Our findings support those of other studies that have found elevated CORT levels associated with infection [9], [10], [13] and associated with anthropogenic stressors such as pesticides and fungicides [33]–[35] suggesting that CORT plays a role in manipulating immunity. We also found that species with different *Bd* lineages showed different stress levels. While it is not known how elevated CORT along with infection by *Bd* affects survival and metamorphosis, this study provides a basis for a more full understanding of the complicated interaction between *Bd* infection and stress in amphibians. Acquiring these data using water-borne hormones from individuals is a significant advance for conservation biology of amphibians as our method does not require sacrificing individuals, provides insight into the health of individuals/populations, and can be used to reassess their health at a later point to examine temporal patterns. In sum, this new technique will be useful in studies of amphibian physiology, immunology and conservation and disease ecology.

## References

[1] Stuart SN, Chanson JS, Cox NA, Young BE, Rodrigues ASL, et al (2004) Status and trends of amphibian declines and extinctions worldwide. Science 306: 1783–1786. 15486254 10.1126/science.1103538

[2] Fisher MC, Garner TWJ, Walker SF (2009) Global emergence of *Batrachochytrium dendrobatidis* and amphibian chytridiomycosis in space, time, and host. Annual Review of Microbiology 63(1): 291–310. 10.1146/annurev.micro.091208.073435 19575560

[3] Kilpatrick M, Briggs C, Daszak P (2010) The ecology and impact of chytridiomycosis: an emerging disease of amphibians. Trends in Ecology and Evolution 25: 109–118. 19836101 10.1016/j.tree.2009.07.011

[4] Woodhams D, Bosch J, Briggs C, Cashins S, Davis L, et al (2011) Mitigating amphibian disease: strategies to maintain wild populations and control chytridiomycosis. Frontiers in Zoology 8: 8. 21496358 10.1186/1742-9994-8-8 PMC3098159

[5] Ribas L, Li M, Doddington B, Robert J, Seidel J, et al (2009) Expression profiling the temperature-dependent amphibian response to infection by *Batrachochytrium dendrobatidis* . PLoS One 4: e8408. 20027316 10.1371/journal.pone.0008408 PMC2794374

[6] Rollins-Smith LA, Ramsey JP, Pask JD, Reinert LK, Woodhams DC (2011) Amphibian immune defenses against chytridiomycosis: impacts of changing environments. Integrative and Comparative Biology 51: 552–62. 21816807 10.1093/icb/icr095

[7] Rollins-Smith LA (2001) Neuroendocrine-immune system interactions in amphibians - Implications for understanding global amphibian declines. Immunologic Research 23(2–3): 273–280. 11444392 10.1385/IR:23:2-3:273

[8] Sapolsky RM, Romero LM, Munck AU (2000) How do glucocorticoids influence stress Responses? Integrating permissive, suppressive, stimulatory, and preparative actions. Endocrine Reviews 21: 55–89. 10696570 10.1210/edrv.21.1.0389

[9] Dhabhar FS (2002) A hassle a day may keep the doctor away: Stress and the augmentation of immune function. Integrative and Comparative Biology 42: 556–564. 21708751 10.1093/icb/42.3.556

[10] Warne RW, Crespi EJ, Brunner JL (2011) Escape from the pond: stress and developmental responses to ranavirus infection in wood frog tadpoles. Functional Ecology 25(1): 139–146.

[11] Denver RJ (2009) Stress hormones mediate environment-genotype interactions during amphibian development. General and Comparative Endocrinology 164: 20–31. 19393659 10.1016/j.ygcen.2009.04.016

[12] Davis A, Keel M, Ferreira A, Maerz J (2010) Effects of chytridiomycosis on circulating white blood cell distributions of bullfrog larvae (*Rana catesbeiana*). Comparative Clinical Pathology 19: 49–55.

[13] Kindermann C, Narayan EJ, Hero J-M (2012) Urinary corticosterone metabolites and chytridiomycosis disease prevalence in a free-living population of male Stony Creek frogs (*Litoria wilcoxii*). Comparative Biochemistry and Physiology Part A, Molecular & Integrative Physiology 162: 171–176. 10.1016/j.cbpa.2012.02.018 22387450

[14] Garner TWJ, Rowcliffe JM, Fisher MC (2011) Climate change, chytridiomycosis or condition: an experimental test of amphibian survival. Global Change Biology 17: 667–675.

[15] Garner T, Walker S, Bosch J, Leech S, Rowcliffe J, et al (2009) Life history tradeoffs influence mortality associated with amphibian pathogen *Batrachochytrium dendrobatidis* . Oikos 118: 783–791.

[16] Farrer RA, Weinert LA, Bielby J, Garner TWJ, Balloux F, et al (2011) Multiple emergences of genetically diverse amphibian-infecting chytrids include a globalized hypervirulent recombinant lineage. Proceedings of the National Academy of Sciences of the United States of America 108: 18732–18736. 22065772 10.1073/pnas.1111915108 PMC3219125

[17] Savage AE, Zamudio KR (2011) MHC genotypes associate with resistance to a frog-killing fungus. Proceedings of the National Academy of Sciences of the United States of America 108: 16705–16710. 21949385 10.1073/pnas.1106893108 PMC3189034

[18] Tobler U, Schmidt B (2010) Within- and among-population variation in chytridiomycosis-induced mortality in the toad *Alytes obstetricans* . PLoS ONE 5: e10927. 20532196 10.1371/journal.pone.0010927 PMC2880007

[19] Blaustein AR, Gervasi SS, Johnson PTJ, Hoverman JT, Belden LK, et al (2012) Ecophysiology meets conservation: understanding the role of disease in amphibian population declines. Philosophical Transactions of the Royal Society B: Biological Sciences 367: 1688–1707. 10.1098/rstb.2012.0011 PMC3350657 22566676

[20] Scott AP, Ellis T (2007) Measurement of fish steroids in water - a review. General and Comparative Endocrinology 153: 392–400. 17188270 10.1016/j.ygcen.2006.11.006

[21] Scott AP, Hirschenhauser K, Bender N, Oliveira R, Earley RL, et al (2008) Non-invasive measurement of steroids in fish-holding water: important considerations when applying the procedure to behaviour studies. Behaviour 145: 1307–1328.

[22] Gabor CR, Bosch J, Fries J, Davis D (In press) A non-invasive water-borne hormones assay for amphibians. Amphibia-Reptilia.

[23] Bosch J, Martinez-Solano I, Garcia-Paris M (2001) Evidence of a chytrid fungus infection involved in the decline of the common midwife toad (*Alytes obstetricans*) in protected areas of Central Spain. Biology Conservation 97: 331–337.

[24] Fisher M, Bosch J, Yin Z, Stead D, Walker J (2009) Proteomic and phenotypic profiling of an emerging pathogen of amphibians, *Batrachochytrium dendrobatidis*, shows that genotype is linked to virulence. Molecular Ecology 18: 415. 19161465 10.1111/j.1365-294X.2008.04041.x

[25] Walker S, Bosch J, Gomez V, Garner T, Cunningham A, et al (2010) Factors driving pathogenicity versus prevalence of amphibian panzootic chytridiomycosis in Iberia. Ecology Letters 13: 372–382. 20132274 10.1111/j.1461-0248.2009.01434.x

[26] Gosner K (1960) A simplified table for staging anuran embryos and larvae with notes on identification. Herpetologica 16: 183–190.

[27] Earley RL, Hsu Y (2008) Reciprocity between endocrine state and contest behavior in the killifish, *Kryptolebias marmoratus* . Hormones and Behavior 53: 442–451. 18191133 10.1016/j.yhbeh.2007.11.017

[28] Boyle D, Boyle D, Olsen V, Morgan J, Hyatt A (2004) Rapid quantitative detection of chytridiomycosis (*Batrachochytrium dendrobatidis*) in amphibian samples using real-time Taqman PCR assay. Dis Aquatic Organisms 60: 141. 10.3354/dao060141 15460858

[29] Walker S, Bosch J, James T, Litvintseva A, Valls J, et al (2008) Invasive pathogens threaten species recovery programs. Current Biology 18: R853–R854. 18812076 10.1016/j.cub.2008.07.033

[30] Jaudet GJ, Hatey JL (1984) Variations in aldosterone and corticosterone plasma levels during metamorphosis in *Xenopus laevis* tadpoles. General and Comparative Endocrinology 56(1): 59–65. 6489739 10.1016/0016-6480(84)90061-3

[31] Kikuyama S, Kawamura K, Tanaka S, Yamamoto K (1993) Aspects of amphibian metamorphosis: hormonal control. International Review of Cytology - a Survey of Cell Biology, Vol 145: 105–148. 10.1016/s0074-7696(08)60426-x 8500980

[32] Glennemeier KA, Denver RJ (2002) Role for corticoids in mediating the response of *Rana pipiens* tadpoles to intraspecific competition. Journal of Experimental Zoology 292: 32–40. 11754020 10.1002/jez.1140

[33] Hayes TB, Case P, Chui S, Chung D, Haeffele C, et al (2006) Pesticide mixtures, endocrine disruption, and amphibian declines: Are we underestimating the impact? Environmental Health Perspectives 114: 40–50. 10.1289/ehp.8051 PMC1874187 16818245

[34] Martin LB, Hopkins WA, Mydlarz LD, Rohr JR (2010) The effects of anthropogenic global changes on immune functions and disease resistance. In: Year in Ecology and Conservation Biology Ostfeld RS, Schlesinger WH, editors. 2010: 129–148. 10.1111/j.1749-6632.2010.05454.x 20536821

[35] McMahon T, Crumrine P, Halstead N, Johnson S, Raffel TR, et al (2011) The fungicide chlorothalonil is nonlinearly associated with corticosterone levels, immunity, and mortality in amphibians. Environmental Health Perspectives 119: 1098–1103. 21463979 10.1289/ehp.1002956 PMC3237349

